# Relapse among MHCUs after a Short-Term Admission in an Acute Psychiatric Unit: Primary Caregivers’ Perspective

**DOI:** 10.3390/ijerph20021384

**Published:** 2023-01-12

**Authors:** Nelson Raluthaga, Hilda N. Shilubane, Mygirl Pearl Lowane

**Affiliations:** 1 Department of Advanced Nursing Science, University of Venda, Private Bag X5050, Thohoyandou 0950, South Africa; 2 Department of Public Health, Sefako Makgatho Health Sciences University, P.O. Box 215, Medunsa, Pretoria 0204, South Africa

**Keywords:** acute mental health, relapse, primary caregiver, acute psychiatric unit, deinstitutionalization

## Abstract

South Africa has taken initiative to strengthen its mental health system, by improving the Mental Health Care Act 17 of 2002 which proclaims that mental healthcare users (MHCUs) can be treated in communities and homes. Due to short-term hospitalisations for acute MHCUs and advocacy for community-based care, families play a significant role in providing care to severe mental healthcare users. The objective of the study was to explore primary caregivers’ perspective regarding the relapse of MHCUs following a short-term admission in acute psychiatric units. A qualitative explorative design was used. In-depth individual interviews were conducted with 18 primary caregivers whose family members were readmitted to four hospitals with units designated for acute MHCUs in Limpopo. NVivo computer software version 11 was used to analyse data. The findings are that MHCUs deny the mental health condition. Mental illness is considered a short illness that can be cured, which shows misconceptions about self-mental health conditions. Refusal of direct observed treatment support also emerged; hence, it is difficult for caregivers to identify if the patient is taking the correct doses or not taking the medication at all. Perceived wrong beliefs about mental illness can affect the patient’s desire to seek proper management and it can be damaging in many ways. Drugs and alcohol abuse makes MHCUs display disruptive behaviours and contribute to treatment non-adherence resulting in caregivers becoming reluctant to be around them. In conclusion, mixing traditional and faith-based mental healthcare practices as reported by primary caregivers can mean that tailor-fabricated culture-specific mental healthcare is required.

## 1. Introduction

Mental illness in South Africa was found to be highest in the African region, with the highest prevalence of chronic mental disorders among mental healthcare users (MHCUs) [1,2]. Many MHCUs are treated as voluntary or involuntary inpatients in the hospital setting before being discharged to a primary health care or home environment [3]. Voluntary and involuntary acute mental healthcare users require short-term treatment therapy during hospitalisation until they are deemed to have recovered enough to be treated as an outpatient [4]. The admission period varies from patient to patient depending on the response to the therapy.

Because some mental illnesses allow MHCUs to be managed at home, treatment interruption is the most common cause of relapse among MHCUs [5,6]. Relapse in a mental healthcare user is defined as “a step of regression from a particular level of stability” [7,8] and occurs when a patient develops mental health illness symptoms after recovery [7]. Relapse in MHCUs contributes to the global burden of mental illness and creates barriers to effective and successful recovery and rehabilitation [7]. Patients who relapse are admitted to an acute phase unit in the hospital and stabilized before being discharged as out-patients under the care of primary caregivers.

The transition from acute units to home is regarded as a critical transition process [9]. Furthermore, the transition from hospital to home care is difficult and complicated [9], because MHCUs face additional risks to their emotional well-being [10], resulting in readmission. According to some studies, entering and exiting an acute in-patient unit is a devastating, chaotic, emotional, and stressful transition point of care in mental healthcare services [10,11,12]. Acute MHCUs are said to be caught in a “revolving door” because they frequently transition between inpatient hospitalization and community care, only to return to hospitalization within a short period of time [13]. Some studies found medication management, a worse mental health state as a result of the distress, and an increased risk to oneself and family relationships [14].

Despite all the challenges, South Africa has taken initiative to strengthen its mental health system, by improving the Mental Health Care Act 17 of 2002 [1,15] which proclaims that MHCUs can be treated in communities and homes and is called deinstitutionalization of mental healthcare program [1,2,15]. Many mental healthcare users require a substantial amount of help from primary caregivers. Due to short-term hospitalisations for acute MHCUs and advocacy for community-based care, families play a significant role in providing care to severe mental healthcare users [16,17].

Since the literature highlights that there is a high percentage of mental healthcare users discharged from the hospital to their family members [18,19], it is critical to assess and address the families’ perspectives, concerns, desires, and needs through therapists or case managers for their MHCU relatives. As a result of the deinstitutionalization movement, families have all too often become de facto therapists or case managers for their MHCU relatives [20]. The family members are found to be primary resources for MHCUs, even after a long stay in the hospital, regardless of whether they stay together or not [21]. In Addition, mothers are usually found to be the primary caregivers of MHCUs [20].

Many families always strive to maintain contact and involvement with their loved ones during and post-discharge. They can go above and beyond by providing material and financial resources to ensure that their relatives recover. Most studies have focused on the barriers faced by caregivers or families in caring for MHCUs following a long-term hospitalisation. There is scant research on the causes of relapse among MHCUs shortly after discharge from acute psychiatric units within the South African context, hence the present study seeks to explore in detail what causes MHCUs to relapse shortly after discharge from acute psychiatric units, as observed by primary caregivers in Limpopo Province.

## 2. Materials and Methods

### 2.1. Research Design and Setting

This is a qualitative, exploratory, descriptive, and contextual research to explore primary caregivers’ perspectives regarding relapse of MHCUs following a short-term admission in acute psychiatric units. Four hospitals with units designated for acute MHCUs were purposely selected in Limpopo province.

### 2.2. Study Population and Sampling Procedure

The primary caregivers of MHCUs admitted to the hospital at the time of data collection comprised the study population. The participants were conveniently selected as they came to see their loved ones admitted to short-term therapy in psychiatric units. Those who met the inclusion criteria were identified with the assistance of the help desk psychiatric unit nursing staff. Caregivers were chosen if they were at least 18 years old and capable of providing informed consent. Forty primary caregivers, 10 per hospital, were contacted and recruited to form part of the study as they entered the short-term or acute unit. However, data saturation from 18 participants was declared because the successive discussions were yielding no more new knowledge that promotes a better understanding of the phenomenon under investigation in this study.

### 2.3. Data Collection Tool

Data were gathered using a semi-structured interview guide. The interview guide asked participants for information about MHCU relapse after a short-term stay in acute psychiatric units. The development of the interview guide was informed by previous research on family perspectives related to caring for mental healthcare users [19]. The interview guide question was structured in English, then translated into Tshivenda, Xitsonga, and Sepedi, because these are the most spoken languages in the study setting. The data collecting tool was pre-tested with four primary caregivers of MHCUs who shared a similar background as the participants.

### 2.4. Data Collection Procedure

Arrangements were made with caregivers who consented to be part of the study. Some caregivers agreed to be interviewed on the same day after they were informed about the study. However, some caregivers agreed to be interviewed on their next visit to the hospital. The locations and times for the interview were then arranged. Before the beginning of the in-depth individual interviews, informed consent was obtained. Participants were informed that their participation was entirely voluntary and that they could opt out of the study at any time during the interview. To ensure privacy and confidentiality, the interviews were held in a room provided by the unit manager.

The interviews were conducted from July to September 2020, and each interview session lasted 30 to 45 min. Interviews were conducted in a language the participant preferred. Using the preferred language allowed the participants to speak freely and provide a rich response about their view on why mental healthcare users relapse after being discharged from the hospital. To gain a better understanding of the phenomenon in question, follow-up questions were asked. With the participants’ permission, the interviews were recorded using a voice recorder. The eighteen participants interviewed constituted the sample size of this study. Brief demographics were collected from each participant at the end of the interview to provide context to the data collected.

### 2.5. Data Analysis

The voice recordings were transcribed verbatim firstly in Tshivenda, Xitsonga, and Sepedi and later interpreted into the English language by the principal investigator and the co-authors. All the authors were involved in comparing translated transcripts to voice recordings for accuracy. NVivo computer software version 11 was used for data analysis, and a general inductive approach was used. The predefined themes were investigated, and subthemes were derived from the main themes using an inductive process. As the coding progressed, a ‘tree structure’ emerged, with themes and subthemes interconnected. The findings are organised around these themes and subthemes.

### 2.6. Trustworthiness

The four concepts from Lincoln and Guba (1985) namely credibility, dependability, confirmability, and transferability were used to maintain trustworthiness [22]. To ensure credibility the researcher created a trusting relationship with the participants, whereby participants were free to express themselves in their vernacular and had prolonged engagement with the participants during the interview sessions. To ensure transferability, the selected participants were representative of the population and its characteristics. In this study, dependability was achieved through the use of a voice recorder and field notes during data collection to ensure accuracy and the researcher kept hard copies of transcripts, observation, and field notes to ensure an audit trail. Furthermore, confirmability was ensured by recording the true responses of the participants. This was achieved by triangulation using a voice recorder, observation, and field notes.

### 2.7. Ethical Considerations

The University’s Research Ethical Committee granted approval for the study (Ref: SHS/19/PDC/43/0811). Permission to conduct the study was granted by the department of health, Limpopo Province, and all the selected hospitals. All the participants were informed that their participation was voluntary and about the confidentiality of the data. Informed consent was obtained before beginning the interviews. Pseudonyms were used to protect the participants’ identities and to ensure that the information shared was not at any point linked to any of them.

## 3. Findings

### 3.1. Demographic Characteristics of Participants

The sample consisted of 18 primary caregivers of MHCUs readmitted to the acute unit after hospitalisation. Their ages ranged from 24 to 68 years. Most primary givers, 13/18, were ever married, and five of them never married. Among those primary caregivers, only six were males compared with their counterparts who were 12 females. The majority, 12/18, were unemployed and amongst them, nine were depending on social grants. Six of them were employed, and two reported that they are self-employed. The household income of primary health caregivers ranges from as little as R 800 to R 3500 per month. However, three of those working participants reported an income of more than R 3500. Fifteen of the primary caregivers attended school, whereas three of them never attended school. Eleven primary caregivers were living with the patients, whereas seven were not staying with the patients directly, but having contact with the patient daily when discharged from the hospital. This section is presented in Table 1, which provides a concise description of the sociodemographic characteristics of the primary caregivers without their interpretation.

### 3.2. Findings That Emerged from Data Analysis

Five main themes and six subthemes emerged from the analysis of data collected from primary caregivers (Table 2).

#### 3.2.1. Misconception about the Self-Mental Health Condition

The participants noticed a misconception about self-mental illness. The majority of them reported that when MHCUs were discharged, they mentioned that it was only a short illness and that they were cured. They attributed this behaviour to MHCUs’ misconceptions about self-mental health conditions. Some participants stated that they believe what the MHCU was saying because they too have a limited understanding of the condition. This main theme caused two subthemes, which are discussed below:

##### Denial of Mental Health Condition

Denial of mental health condition was among MHCUs. Thirteen participants mentioned that their loved ones stopped taking the psychiatric medication immediately after being discharged from the hospital.

“The problem is that the MHCU does not think that he needs the medication. He thinks he is not mentally ill. I tried to force him to take the mental health tablets but refused and reported that he will take only tablets for headaches”.

(P3, 38 years old sister)

##### Perceived Cured

Participants reported that most of their MHCUs were cured after discharge, as they (MHCUs) reported having no symptoms anymore. Hence MHCUs stop treatment and interrupt going to medical follow-ups. The following was reported:

“She told me that she feels better and that there is no need to take the medication anymore”.

(P13, 44 years old mother)

One participant reported that:

“My son believes that he is fine, and no longer sick. We were so happy to see him in that state, and I did not even bother to ask him about his medication. But I have observed that he is not taking the medication as instructed anymore”.

(P15, 61 years old father)

Another participant reported the following:

“He does not take his medication anymore, because he says he is okay. I do not know if I can get people who can talk to him about the medication so that he can understand his medical condition. We always talk to him about medication, but he undermines me. He says I must tell those who are mentally ill to take their medication, not him”.

(P2, 59 years old grandmother)

#### 3.2.2. Barriers to Supervision

Denied supervision by MHCUs was one of the factors reported by the participants. Participants reported that most of the patients that were relapsing after discharge were those who lack supervision. Two subthemes emerged and are discussed as follows:

##### Refusal of Direct Observation Treatment Support by Caregivers

Refusal of direct observed treatment support was found to be a factor resulting in patients’ relapse. Though stable after discharge, most MHCUs need someone to observe them when taking their medication. Participants reported that most MHCUs do not want to be given treatment by their family members, as some of them felt in control of their condition. One participant reported that MHCUs felt that their autonomy and privacy were violated when directly supervised. Hence, it is difficult for caregivers to identify if the patient is taking the correct doses or not at all. This was expressed in the following quotes:

“He refused to be given treatment by me, indicating that he will take it on his own time. Two weeks later, I started to suspect that something was wrong. I went to count his pills the time he went out, only to find that he still had a lot of supplies. I observed that where he was supposed to take two per day, he was taking one tablet instead”.

(P9. 49 years old aunt)

One participant reported the following:

“I am scared to remind him to take his medication because he gets angry and becomes verbally aggressive whenever I remind him. He swears at me, saying why I treat him like a child. So, I let him keep his medication, I am sure he does not take it the way they told us at the hospital because his behaviour has changed”.

(P3, 38 years old aunt)

Three participants mentioned it was difficult for them to assist MHCUs because they attack them when engaged with them. As a result, participants affirm that most of the time MHCUs were not supervised when taking their medication.

“He does not want us to get involved in his medication. He locks his room and fights with us when we ask if he took his medication. I think he undermines me because I am a woman. Sometimes he looks well, and the other time looks like he has relapsed. I wish I had a male person in the house who would force him to take treatment”.

(P6, 53 years old mother)

##### Leaving Alone after Discharge

Though MHCUs were mostly discharged under the care of primary caregivers, this study found that there are MHCUs who went to their respective areas after discharge. Seven participants reported that they are not staying with the patient in the same home but visit often. This posed a challenge to participants because some medications are taken at night. Two participants reported that they sometimes did not find the MHCU when visiting. Others reported the following:

“I think this time after discharge, I will insist that he comes to my place. There is no one in his parents’ place as all are deceased..... I thought he will be able to manage his condition since he is an adult and I was mistaken”.

(P16, 65 years old grandfather)

“I think it was the biggest problem to leave him alone. Even though we suggested that he must come and stay with us. He would come for one week and later return to his place. Some weeks later the neighbours would call to inform us that he relapsed again”.

(P4, 50 years old uncle)

#### 3.2.3. The Perceived Belief about Mental Illness

Perceived wrong beliefs about mental illness can affect the patient’s desire to seek proper management and it can be damaging in many ways. This study learned from caregivers that MHCUs have wrong information about their condition, and this might be a factor in relapse. Participants reported that some patients refrain from adhering to treatment plans because of thoughts of witchcraft and seeking alternative therapy for their condition. Hence these two subthemes emerged from this main theme.

##### Thought of Witchcraft

Some participants strongly agree that the issue of witchcraft affects MHCUs from obtaining the help they need. Two participants reported that some MHCUs hide the status because they think their first episode was the result of witchcraft. They further indicated that some MHCUs fear how people will view them if they come forward with their illnesses.

“My mother thinks she is sick because one in the family has bewitched her. She does not want to talk about the condition or treatment. I think this increases her stress level because she is always angry and bitter”.

(P11, 24-year-old daughter)

On contrary, one participant mentioned that:

“People thought that psychotic patients are witches. With this myth, some MHCUs avoid their clinic follow-up visit for treatment collection because they are afraid of going public with that stigma attached to them”.

(P17, 27 years old sister)

Some participants reported that some MHCUs avoid treatment due to the fear of being bewitched. They also stated that the thought left patients struggling on their own and with negative attitudes toward mental illness, as well as negative attitudes toward the internalization of mental healthcare services. Participants believe that these situations aggravate the problem, hence MHCUs relapse.

##### Seeking Alternative Therapy

Seeking alternative therapy was reported by the majority of the participants. This subtheme emerged as a follow-up of the above subtheme (thought of bewitched) during probing. Participants reported that some MHCUs were taken to traditional health practitioners (Sangoma) to receive other forms of treatment.

“My sister was taken to the “Sangoma” by my aunt after she was discharged from the hospital. She was told to use the traditional medicine to bathe with to remove the dark cloud hanging on her”.

(P14, 38 years old brother)

Three participants reported that religious belief may be the contributory factor inhibiting MHCUs to adhere to their treatment therapy. This suspicion was supported by the following quote:

“My daughter refuses to take medication, as she says that the pastor in the church thinks she has “demons” [evil spirits]. She says the pastor prayed for her and instructed her to stop taking the medication”.

(P16, 58 years old mother)

#### 3.2.4. Change in Mental Health Treatment

Some participants reported that the non-adherence to medication by MHCUs was related to changes made by doctors to their medications. MHCUs become used to their medications, for instance, the number and colour of the tablets. Changes in medication type and packaging leads to non-compliance by MHCUs. The following quotes attest to this:

“They changed his injection and also added tablets. Since he was put on the new injection, his re-admissions are frequent. When he was on the previous one, he used to stay for a year without re-admission. He was used to it”.

(P1, 59 years old grandmother)

“She was taking the pink tablets for almost three years now. The problem started when the hospital ran out of pink tablets. They gave her white tablets in a box, unlike the previous one, which was in sachets. Those tablets work the same way, but she says hers are pink, not white. She believes they gave her the wrong tablets, and she does not take them”.

(P8, 60 years old grandmother)

#### 3.2.5. Exposure to Behaviour That Aggravates the Mental Health Condition

The study found that MHCUs returned to old behaviours immediately after discharge. Substance and alcohol use was reported by the participants to be the factor causing non-adherence to medication by MHCUs. It was found during the interviews that caregivers wished that their MHCUs would not use substances as it significantly impaired their judgments and aggravated their mental health condition. This is what the participants had to say about the use of substances:

“It does not help to drink alcohol while one is on treatment because that alcohol dilutes the tablets. Sometimes he comes home late at night and gets drunk. I cannot force him to take medication”.

(P7, 59 years old grandfather)

Two participants reported that they felt helpless because their patients became rebellious under the influence of substances and alcohol.

“He wakes up early in the morning and goes to his friends. They smoke dagga the whole day. Once he is intoxicated with dagga, he does not listen to anybody. I am afraid of him because he is aggressive. Hence most days he missed the treatment”.

(P5, 47 years old mother)

“Alcohol use makes him stop taking treatment. If you dare tell him about medication, he will swear at you with vulgar words. He takes treatment whenever he likes. I just keep quiet and let him do as he pleases”.

(P6, 53 years old father)

“I do not know what we can do to make him take his treatment because that is the only thing that can save him from relapsing. If he can stop drinking this ‘mahafhe’ (home-brewed liquor), I believe everything would be fine. Liquor is the main problem”.

(P10, 49 years old mother)

## 4. Discussion

The findings from this study highlight the primary caregivers’ perspectives on what causes MHCUs to relapse shortly after being discharged from a mental health unit. Several factors were observed by the primary caregivers as the main reasons for MHCUs to redevelop psychiatric symptoms after being stabilised in hospitalisation. Although several studies have investigated factors associated with the occurrence of psychiatric symptoms after discharge, few studies have examined whether misconception about self mental health conditions is the cause of relapse among MHCUs [1,2,7,23]. Denial and perceived cure by MHCUs are the major barriers to complying with treatment. This study found that MHCUs who perceived themselves as cured or mistakenly diagnosed were found to reject taking their medication, even if the primary caregivers intervened. The findings agree with [24], who found that family members often struggle to persuade MHCUs to take treatment as they have difficulty acknowledging that they are mentally ill. This was supported by a study conducted by [2], which highlighted that denial of mental health conditions and perceived cure is very much common among MHCUs who have poor health literacy regarding their condition, hence it results in poor health outcomes.

Supervision of MHCUs is of particular concern because it affects the rate of early relapse after discharge from the hospital. This study found that there is a gap in supervision issues between patients and caregivers that needs to be addressed before the patients are deinstitutionalized. MHCUs need consistent supervision. However, it is not always possible because primary caregivers are denied supervision. The primary caregivers need to take the responsibility of continuous supervision regardless of whether patients are feeling better or not. Moreover, MHCUs must not stay alone because they need supervision regarding the taking of medication. Staying alone can add to the stress that might increase the risk of relapse into psychotic disorders [25]. Prior to deinstitutionalisation, for people who were unable to care for themselves if they were left alone, other measures such as linking MHCUs to social services can be implemented to supplement the primary caregiver’s availability. A psychiatric healthcare professional should discuss the role of primary caregivers with the patient prior to discharge. The primary caregivers reported the issue of witchcraft and sought alternative treatment in the context of mental illness. This implies that MHCUS and some primary caregivers might have a poor understanding of the subject of mental illness and that it requires medical therapy. It was highlighted in the literature that there is a gap in mental health treatment, especially in underdeveloped countries [26]. In the same context, there is a clear suggestion that MHCUs might also be individuals seeking or relying on alternative therapies from traditional healthcare practitioners or faith-based practitioners [26]. This might also be perpetuated by the thought of witchcraft as reported by the caregivers who participated in this study. Similar studies support that most patients living with mental illness utilise traditional health practitioner services, which might be perhaps the belief regarding the cause of their mental illness, and the reasons for not utilising mental healthcare services remain a mystery [27]. According to the findings of this study, the prevalence of informal mental medical practices among patients living with mental illness contributes to delays in seeking mental healthcare services in some cases. The mixing of mental health treatment practices was found to be a contributory factor compromising compliance with therapy in this study. Hence, as a result, MHCUs fall into relapsing at the end of the day.

Change in mental health treatment was identified by the primary caregivers as a factor in the refusal of mental health treatment. Changes in the medications cause confusion among MHCUs and led to non-adherence. Though the treatment might have the same effects, things like pill colour and container play a crucial role in medication adherence [28]. Mental health treatment, specifically antipsychotic treatments reduce the severity of mental illness and delay relapse [29,30]. It is empirical to empower people living with mental illness so that they become knowledgeable regarding their treatment and actively manage their mental illnesses. Some authors argue that MHCUs who interrupt treatment one to ten days after discharge increase the risk of re-admission [30,31].

The finding in this study demonstrated that primary caregivers observed MHCUs subjecting themselves to behaviour that aggravates the mental health condition. The use of illicit drugs and alcohol abuse were identified to be the strongest predictors of treatment interruption [32]. Substance and alcohol abuse are highly prevalent disorders among MHCUs worldwide [33]. MHCUs suffer double the burden to their illness due to additional substance and alcohol use. Primary caregivers in the study conducted in Ghana felt that MHCUs aggravate their mental conditions through continued substance use [34]. Alcohol use is regarded as an undertreated mental disorder mostly occurring in developed countries, and individuals demonstrate poor judgement toward the use of it, despite the damaging effects on their health [35]. Consequently, there is a higher association between treatment non-adherence and substance abuse as substances destabilise the mental state of MHCUs [36,37]. Drugs and alcohol abuse makes MHCUs display disruptive behaviours, as a result, caregivers become reluctant to be around them.

Mental health policies, guidelines, and practices for mental healthcare proposed by the World Health Organization change from time to time [38]. Prolonged stays at psychiatric hospitals have been replaced with community mental health services and short-term stays in mental health units at general hospitals [39], which decreases the admission period and reduces the provision of good care [25]. Although short-term admission to an acute psychiatric unit has been found to prolong long-term hospitalization [40,41], deinstitutionalisation reduces rehabilitation, provision of adequate time for planning discharge, and a period of evaluation.

## 5. Conclusions

In South Africa, there is a scarcity of data on the relapse of mental healthcare users following a short-term admission to an acute psychiatric unit. Regardless, the current study aimed to provide insights into Primary caregivers’ perspectives on MHCU relapse. The current findings suggest that identifying MHCU characteristics associated with the risk of relapsing after hospitalization is critical for proper deinstitutionalization planning. This study demonstrates that patients themselves are the ones who can prevent sudden relapses if they comply with mental health therapy. In addition, patients demonstrated misconceptions about their own mental health condition and refused direct observed treatment support, making it difficult for caregivers to determine whether or not the patient is taking the medication at all. This necessitates the empowerment of MHCUs by primary caregivers. False beliefs about mental illness can prevent patients from seeking appropriate treatment. A public awareness campaign should be launched to educate patients about their condition. Drugs and alcohol abuse makes MHCUs display disruptive behaviours, which leads to caregivers’ reluctance to be around them. Therefore, the public should be empowered about the dangers of substances to MHCUs which may help them refrain from providing them substances.

Combining traditional and faith-based mental healthcare practices may indicate the need for culture-specific mental healthcare. Collaboration and coalition building between communities and healthcare providers can be advantageous. Healthy lifestyle choices are encouraged by creating healthy communities. It is imperative that future quantitative studies be conducted to ensure the representativeness and generalizability of the findings.

## Figures and Tables

**Table 1:** Sociodemographic characteristics of the primary caregivers of MHCUs sample for this study.

Variable	Sub-Category	Frequency
Gender	Female	12
	Male	6
Age category in years	20–39	4
	40–49	5
	50–60	7
	61 and above	2
* Marital status	Ever married	13
	Never married	5
* Education status	* Ever attended school	15
	Never attended school	3
Household income	Less than 1000	4
	1000 to 5000	11
	5001 and above	3
Relationship with the MHCU	Mother	9
	Father	2
	* Sibling	3
	* Relative	4
Living in the same household with the MHCU	Yes	11
	no	7

* Marital status: ever married refers to married, divorced, or widow; never married refers to single or cohabiting. * Education Status: ever attended school refers to attended primary, secondary, and tertiary level of education. * Sibling: biological, stepsister, or brother. * Relatives: any close relatives including an aunt, uncle, and grandparent.

**Table 2:** Summary of themes and subthemes.

Themes	Sub-Themes
Misconception about the self-mental health condition	☐ Denial of mental health condition ☐ Perceived cured
Barriers to supervision	☐ Refusal of direct observation support by caregivers ☐ Leaving alone after discharge
The perceived belief of mental health illness	☐ Thought of witchcraft ☐ Seeking alternative therapy
Change in mental health treatment	
Exposure to behaviour that aggravates the mental health condition	

## Data Availability

Data presented in this study can be obtained from the first author upon reasonable request.
